# Stroke care networks and the impact on quality of care

**DOI:** 10.1007/s10729-021-09582-0

**Published:** 2021-09-25

**Authors:** Jan Schoenfelder, Mansour Zarrin, Remo Griesbaum, Ansgar Berlis

**Affiliations:** 1 Department of Health Care Operations/Health Information Management, Faculty of Business and Economics, University of Augsburg, Universitätsstraße 16, 86159 Augsburg, Germany; 2 Department of Diagnostic and Interventional Neuroradiology, University Hospital Augsburg, Stenglinstr. 2, 86156 Augsburg, Germany

**Keywords:** Stroke care, Simulation modeling, Clustering, Network design, Operations research, Operations management

## Abstract

Lack of rapidly available neurological expertise, especially in rural areas, is one of the key obstacles in stroke care. Stroke care networks attempt to address this challenge by connecting hospitals with specialized stroke centers, stroke units, and hospitals of lower levels of care. While the benefits of stroke care networks are well-documented, travel distances are likely to increase when patients are transferred almost exclusively between members of the same network. This is particularly important for patients who require mechanical thrombectomy, an increasingly employed treatment method that requires equipment and expertise available in specialized stroke centers. This study aims to analyze the performance of the current design of stroke care networks in Bavaria, Germany, and to evaluate the improvement potential when the networks are redesigned to minimize travel distances. To this end, we define three fundamental criteria for assessing network design performance: 1) average travel distances, 2) the populace in the catchment area relative to the number of stroke units, and 3) the ratio of stroke units to lower-care hospitals. We generate several alternative stroke network designs using an analytical approach based on mathematical programming and clustering. Finally, we evaluate the performance of the existing networks in Bavaria via simulation. The results show that the current network design could be significantly improved concerning the average travel distances. Moreover, the existing networks are unnecessarily imbalanced when it comes to their number of stroke units per capita and the ratio of stroke units to lower-care hospitals.

## Highlights

- We provide the first study that addresses stroke networks’ adverse effect on the quality of care regarding stroke patients’ travel distances.
- We apply a combination of clustering, optimization, and simulation in our study.
- We evaluate the expected travel distances within the existing stroke networks in Bavaria, the largest state in Germany, and compare them to alternative optimized network designs.
- Our study highlights the negative impact on patient care when stroke care networks employ a policy of transferring patients between hospitals of the same network (almost) exclusively.

## Introduction

Stroke is a common and serious disease that can have lasting health consequences. It is one of the leading causes of death in many countries (e.g., the second most frequent cause of death in Germany) and is considered one of the leading causes of disability in adults [28]. According to an estimation of the World Health Organization (WHO), 17.9 million people (the number 1 cause of death globally) died because of cardiovascular diseases around the world every year, 85% of them are due to strokes and heart attacks [47]. Referring to the Federal Health Monitoring^1^ report, cerebrovascular diseases treated in hospitals, including stroke, decreased until 2005, but have been rising steadily after that [26, 33]. While mortality rates have been falling steadily in recent decades, the number of incidents that can be linked to strokes is increasing ([17], p. 44). Since 2005, the number of cerebrovascular diseases has increased by about 3200 annually. In 2013, 58,556 patients (35,389 women and 23,167 men) died because of cerebrovascular diseases in Germany [17, 26].

This represents an increasing financial burden for the government and the health insurance funds. In 2015, the costs for diseases of the cardiovascular system categorized according to ICD-10 were more than 46,000 million euros, putting them at the top of the list of diseases in Germany [44]. There are 322 of these specialized stroke units in Germany. 57 of them are located in Bavaria (Deutsche [39]). While stroke patients traditionally receive a mixed treatment of general medicine, neurology, and care for the elderly, there have been Stroke Units (SUs) that consist of an interdisciplinary team of doctors and have a sole focus on the treatment of strokes [27].

Due to limited financial and human resources, many smaller regional hospitals lack the necessary equipment and neurological presence [11] to adequately diagnose and treat stroke patients. Especially in rural regions, the supply of SUs is often insufficient. Since stroke symptoms can be easily identified audio-visually, for example by video examination, stroke is suited well for telemedicine cooperation between hospitals [4, 13].

Over the last few decades, telemedicine networks have been established that cooperate specifically in the treatment of strokes [19, 25, 48]. Using a telemedicine approach in stroke care enables the coverage of large areas of low population density and increases the use of evidence-based therapy [20]. Regional differences in stroke care for patients in hospitals without their stroke units can be reduced by better accessibility of stroke units [3]. In these networks, there are three hospital types including stroke centers (SCs) and stroke units (SU) acting as hubs and telemedicine-assisted stroke ready hospital (TSRH) units [20] representing spokes.

According to the European Stroke Organisation (ESO), hubs cover the full pathway of a stroke center containing endovascular, intravenous thrombolysis (IVT), and neurosurgical interventions – with the exception that SUs do not perform mechanical thrombectomies (MTs). In the following text, SU (SC) always refers to a stroke unit (stroke center) certified by the German Stroke Society (DSG). TSRHs are capable of managing stroke patients acutely, for instance administering IVT, but they are not responsible to provide further stroke care. Therefore, any admitted patient will need to be transferred from the TSRH to a hub in its network. Accordingly, a collection of SCs, SUs, and TSRHs is to be understood as a network.

More recently, telemedicine networks have evolved into stroke care networks, which refers to the association of lower-care-level hospitals, often located in rural areas, and hospitals equipped with specialized SUs and SCs. On top of providing telemedicine assistance to lower-care level hospitals, they offer additional within-network services such as unified quality management, workshops, and continuing training to all members. A substantial body of research has shown that these services have resulted in improvements in stroke treatment outcomes, e.g. shorter hospital stays, better stroke diagnosis, and earlier patient rehabilitation [40], as discussed in Section 2 below. However, stroke care networks also ensure that stroke patients admitted to one of the collaborating hospitals are rarely transferred outside of the network (compare [15, 34, 49]). If a patient arrives at a spoke hospital in a particular network and needs additional treatment, e.g. MT, they will be transported to a hub within the same network, unless there is insufficient capacity to perform treatment in all potential within-network hubs. In that case, an outside-network hub will be contacted. Accordingly, stroke patients are not transported to the closest SU if it happens to be outside of the stroke care network. Our study assesses the expected increase in travel distances for stroke patients that arises due to this policy across multiple stroke care networks for the region of Bavaria, Germany, where five competing stroke care networks exist.

### Stroke networks with telemedicine in Bavaria

**Fig. 1:**
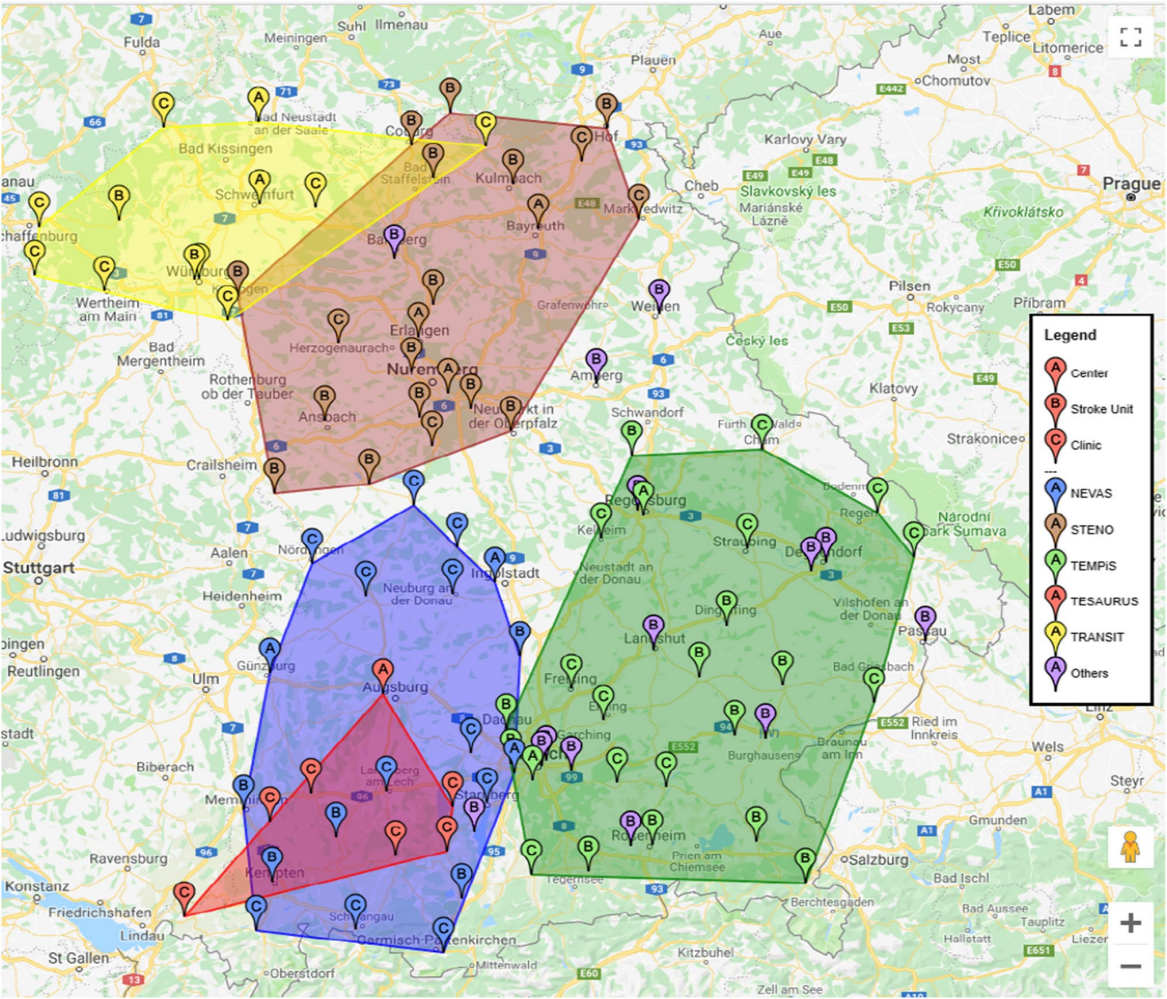
Current stroke networks design in Bavaria

In 1995, the Bavarian State Government started promoting telemedicine expansion. So far, around 60 telemedical projects have been supported with funds of over 13 million euros in Bavaria [6]. Telemedicine makes an important contribution to comprehensive, high-quality medical care in Bavaria in different ways such as teleconsultation, telediagnostics, teletherapy, telemonitoring, and teleteaching. It makes specialist medical knowledge available nationwide for the direct benefit of patients, for example in the emergency treatment of strokes or heart attacks, where every minute counts [31]. The main focus of telemedicine project funding in Bavaria has been on whether the projects directly improve the quality of patient care and to what extent the new technology benefits the patient directly. These projects have focused on classic applications of telemedicine, such as teleconsultations or telemonitoring [6]. However, the current funding is also increasingly targeting widespread applications, the consistent use of standards and increased networking, and in a particular viewpoint, establishing telemedical procedures and establishing medical competence centers. According to the information provided by the German Stroke Society,^2^ there are 57 SUs in the federal state of Bavaria divided into 10 telemedicine, 29 regional, and 18 supraregional stroke units. Five stroke networks containing 83 hospitals exist in Bavaria. 44 of them are SUs (including a single SU located outside of Bavaria), while 14 stroke units do not currently belong to a stroke care network. The TeleMedical Project for Integrative Stroke Care (TEMPiS) is the largest stroke network in Bavaria and one of the leading (tele-)stroke networks in Europe. In the TEMPiS network, 24 regional clinics in Southeast Bavaria receive support from the two stroke centers; Munich-Harlaching Hospital and University Hospital of Regensburg. TEMPiS’s SUs have between 4 and 13 monitored beds. The second-largest network in Bavaria is STENO, a stroke network with telemedicine in northern Bavaria with 3 SCs (University Hospital of Erlangen, Bayreuth Hospital, and Nürnberg Hospital). Its SUs have between 4 and 22 monitored beds. The neurovascular network of Southwest Bavaria or NEVAS is the third-largest Bavarian stroke care network supported by three stroke centers (University Hospital of Munich, Ingolstadt Hospital, and Günzburg District Hospital). The number of monitored beds varies between 4 and 14. TRANSIT in northwest Bavaria is the fourth-largest network and trans-regional network for stroke intervention with telemedicine, which has three stroke centers (Neurological Clinic Bad Neustadt an der Saale, Leopoldina Hospital, and University Hospital of Würzburg). Its SUs have 7 to 10 monitored beds. The TESAURUS telemedicine network (Telemedicine & Stroke Care Augsburg Region & Southwest Bavaria) has existed since January 2010 and has set itself the goal of improving stroke care in the Augsburg area and Southwest Bavaria. The Neurological Clinic of the University Hospital Augsburg as the only stroke center of this network cooperates with currently six connected clinics in the care of cerebrovascular diseases with a focus on the acute treatment of strokes. Figure 1 shows these SUs and partner clinics distributed among the networks. In addition, Table 1 provides more information about these networks and their members.

**Table 1:** Stroke networks in Bavaria

Name	Number of Hubs		Telemedicine-Assisted Stroke Ready Hospital Unit (TSRH)	Total
	Stroke center	Stroke unit		
NEVAS	3	5	11	19
STENO^*^	3	14	4	21
TEMPiS	2	11	11	24
TESAURUS	1	0	6	7
TRANSIT	3	2	7	12

*Note that this network includes an SU which is not located in Bavaria but neighboring Thuringia. For the sake of completeness, this is considered part of the Bavarian network

The rest of this paper is structured as follows:Section 2 presents current developments in the literature by reviewing recent studies conducted on stroke care networks. In Section 3, we present the proposed three-stage method for analyzing the current and alternative network designs. The three stages involve a clustering approach, a mathematical optimization model to redesign the networks, and a simulation used to assess the utilization and possible restrictions of the redesigned networks. Section 4 provides a comprehensive overview of the results. Finally, in Section 5, we summarize our findings and discuss the limitations of the work as well as further research possibilities in this area.

## Literature review

Telestroke was first mentioned in the literature in 1999 to describe the use of telemedicine in providing neurological consultation for stroke in hospitals lacking this level of expertise [29]. Since the late 1990s, several telestroke projects have been developed (mainly) in Western countries to establish efficient diagnosis and intervention for acute ischemic stroke patients. For example, TEMPiS is one of the projects designed to improve the quality of stroke care in a large area of Southeast Bavaria, Germany, that has been comprehensively described by Audebert et al. [5]. Several studies on this project have been conducted from different perspectives. For instance, Müller-Barna et al. [31] conduct an analysis of the first 10 years’ experience. Their results demonstrate that the implementation of stroke care networks increases the number of treated patients with stroke and transient ischemic attack. Also, their results show considerably higher intravenous thrombolysis (IVT) rates, as well as a 40-min (from 80 to 40 min) reduction in door-to-needle and a 30-min reduction (from 150 to 120) in onset-to-treatment times. In another study, by following up 3060 patients, Audebert et al. [5] analyze the long-term effects of acute specialized stroke care with telemedicine support in public hospitals. The results of a multivariable regression analysis show a significant reduction of “death and dependency” at 12 (from 96.5% to 65%) and 30 (from 95.7% to 82%) months. In conclusion, they indicate that the establishment of a stroke care network offers long-term benefits for acute stroke patients. Khan et al. [24] report their experience with a hub-and-spoke telestroke network in the acute care management of stroke patients over two years in Canada. Their results demonstrate that employing telestroke assistance leads to a significant reduction in the number of patients requiring transfer to a tertiary care center (a 92.5% decline; from 144 to 15 at one of the sites). Therefore, telestroke units can provide sustained high-quality care for stroke patients in underserved areas. In other countries, some researchers have focused on telestroke network-specific needs, such as Imai et al. [21] in Japan. They try to determine the requirements of the design and implement telestroke networks and to evaluate the possible effects on the number of thrombolytic therapies. Their study reveals that telestroke may be highly effective in many Japanese stroke hospitals because they do not perform intravenous (IV) tissue plasminogen activator (tPA) in a 24/7 fashion.

Stroke care network design has been examined from technological and medical as well as health economic perspectives. Nelson et al. [32] develop a decision-analytic model for both 90-day and lifetime horizons to conduct a cost-effectiveness analysis of telestroke using audiovisual technology that connects stroke experts to remote physicians at medical units without stroke experts. They show that from a lifetime viewpoint, telestroke networks are cost-effective compared to traditional setups, while also carrying the considerable potential to decrease regional inequalities of acute stroke care provision in the United States. Concerning economic sustainability and cost-effectiveness, Fanale and Demaerschalk [14] review the evidence that supports the reliability and efficacy of telemedicine for diagnosis and acute stroke treatment and propose some strategies for funding the development of a telestroke network.

Over nearly four decades, operations research (OR) models have been utilized to solve problems involving healthcare systems in various ways such as facility location and network design. Ahmadi-Javid, Seyedi and Syam [1] classify different types of emergency and non-emergency healthcare facilities in a location-allocation management perspective and provide ten descriptive dimensions containing multi-period setting, uncertainty issue, objective function, particular input, decision variable, constraints, mathematical modeling approach, basic discrete location problem, solution method, and case study. In another study, Aringhieri et al. [2] conduct a broad literature review on emergency medical services location problems as one of the most important health care services playing a vital role in reducing the mortality rate and morbidity. They identify emerging challenges for future research.

Acute stroke treatment as a complex decision-making problem has great potential for OR to support the effective design and operation of effective and efficient stroke care systems. However, the contribution of OR is so far limited. In this regard, Keshtkaran et al. [23] perform an extensive literature review of validation techniques for healthcare models in acute ischemic stroke. They state that the contribution of OR models to investigate and improve stroke thrombolysis results in a particularly challenging task insofar as model validation is concerned. The multifaceted nature of OR models regularly depends on an extensive diversity of databases and experimental approximations used as inputs [23]. They consider a taxonomy of modeling targets developed by Pidd [36] since they concern modeling purposes. Pidd [36] defines four extensive types of modeling targets containing modeling for decision automation, routine decision support, providing insights, and for investigation and improvement which is the core of discussion in Keshtkaran et al. [23].

Churilov and Donnan [10] review the field of stroke care systems for OR professionals to propose an agenda for future research in the field of designing, planning, and operating stroke care systems. Based on both research and policy contributions reviewed in their study, they present a list of ten comprehensive problem areas that, in the authors’ view, are the most demanding need from the stroke care systems view. Among the areas are the appropriate management of transient ischaemic attacks, stroke unit care, and stroke care networks. They also define four broad groups of potential OR capabilities as operations improvement, economic analysis, public policy, and clinical applications. They finish their discussion of the agenda for OR in stroke care by matching the capabilities to the abovementioned problem areas. In the public policy group, they show that the stroke national and regional planning and network models can be addressed by all ten problem areas.

According to the recommendations provided by Hubert et al. [20] on stroke care networks design in Europe, hospitals/clinics play a part in a stroke care network and should be classified based on criteria such as transportation distance, population density, in-hospital infrastructure, geographic details, and available expert physicians. They conclude that the stroke care network should pledge itself to quality improvement by employing standardized stroke treatment procedures within the network, conducting serious multi-professional training programs, and introducing feedback loops. In the field of telestroke network implementation, French et al. [16] analyze supporting documentation from interviews with key stakeholders of one UK telestroke network and information from existing literature to collate resources and recommendations as well as to identify main challenges in telestroke implementation. In a recent publication, Jauch et al. [22] provide a comprehensive list of recommendations in a single guideline document for healthcare professionals who treat patients with acute ischemic stroke. This guideline uses the American Heart Association format and supports the primary concept of stroke systems in both hospital and pre-hospital settings. To this end, several features from technological, geographical, and demographical viewpoints should be simultaneously considered as barriers to implementing and sustaining telestroke programs. Telestroke network design aims to maintain its logistics and operational sustainability [30] and to engage the commitment of a wide range of stakeholders across multiple organizations [16]. The efficient interactions between hub-and-spoke are more important than the technology for sustaining a telestroke network [42]. Efficient telestroke interactions (for example the interactions between spoke emergency medicine physicians and hub neurologists) successfully fulfill both the economic and clinical requirements of hub-and-spoke hospitals and health care organizations.

This study tries to bridge a part of the gap that exists between OR capabilities and stroke care system requirements and to direct the effort towards important challenges of stroke care network design and operations. Our work aims to analyze the current situation and performance of the existing design of the stroke networks in Bavaria, points out weaknesses in their structures, and presents suggestions for possible improvement of the networks. To this end, we propose an approach containing mathematical modeling, clustering, and simulation. We analyze the performance of both the current and the proposed redesigns of the stroke care networks in Bavaria.

## Methodology

This section presents a framework for an analysis of existing stroke care networks with an application to the state of Bavaria, Germany. The travel distances between hospitals participating in stroke networks should be taken into consideration when patients need to be transferred between spokes and hubs.

In the first stage, the structure of the existing stroke care networks in the German state of Bavaria will be examined. We also identify alternative network designs that are optimized for distances between spokes and hubs. These results serve as a basis for our analyses in the next stages. In the second stage, we propose a mathematical model to assign the stroke units that currently do not belong to any networks to one of the five existing networks by optimizing (minimizing) the average travel distance to all partner hospitals within a network. One important factor that should be considered in the evaluation of stroke care network performance is the available capacity of resources. Three main questions then arise:

1. How well do the current stroke care networks perform in coping with the number of stroke patients?
2. How harmful may existing stroke networks be for providing the best possible stroke care in terms of transfer travel distances?
3. Where are the potential bottlenecks in existing stroke care networks?

For this purpose, we develop a simulation model with the addition of critical capacities in stage three. By simulating the stroke care networks, we incorporate not only the stochastic arrival rates of patients but also the utilization of resources such as staffed beds to identify any resulting bottlenecks in the stroke care networks in Bavaria.

Furthermore, we define three fundamental criteria for assessing network performance to be able to compare the existing network design with the ones optimized for transfer distances. In this way, any shortcomings in the existing network structures and potential improvements can be identified through the proposed alternatives. The first criterion (C1) is the average travel distance from all spokes to their respective nearest hub within a network. These distances are actual driving distances based on the current road infrastructure. In acute stroke care, every minute is critical, as the brain suffers greatly with every minute that goes by without treatment [38]. C1 allows identifying any efficiency gains or losses in network design restructuring, for both individual networks and at the aggregate level. Stroke care network designs have to enable thrombolytic treatment to be administered in community and rural hospitals and to facilitate the appropriate transfer of patients with different situations (e.g., requiring critical care services and neurosurgical or intra-arterial interventions) to a stroke center or hub [18, 43]. Therefore, transportation time is by far the most important criterion in stroke patient treatment and drives the creation of the optimal design of networks. Note that C1 includes only the distance from each spoke to its closest hub, while in reality (and the simulation study) some patients (never more than 20% of the patients in any network in all of our simulation runs) will need to travel to hubs further away due to capacity constraints. Alternative definitions of C1, however, also come with drawbacks. If, for example, C1 included the distances from each spoke to all hubs, it would make the performance criterion unsuitable to compare network designs with different numbers of networks, as the number (and distance) of hubs connected to each spoke would artificially increase when the number of networks decreases. The second criterion (C2) represents the relationship of hubs to the population in each catchment area. This plays an important role in determining the network design performance, as an imbalance between the networks can lead to qualitative losses within an overloaded network. Note, however, that there is no definitively optimal level for C2, i.e. a network does not necessarily perform better the lower C2 becomes. An imbalance between the C2 values of different networks, however, signifies a potential shortcoming in a given network design. The last criterion (C3) is the number of assigned hubs to spoke units within each network. In addition to the population that a network has to supply, the relationship between stroke units and partner clinics within the networks cannot be neglected. Inequality in the distribution of this performance measure between networks can lead to a deterioration in the stroke patient care of the entire region. A network with one hub and 4 spoke units is likely to be less capable of handling the fluctuating volume of patients effectively than a network with two hubs and 3 spoke units. Again, there is no clear optimal value for this measure. The highest possible ratio (only hubs and no spoke units) would not result in an ideal network, as that would eliminate the economic benefits of combining specialized stroke units that assist TSRHs in more remote areas within a stroke care network. Table 2 presents the formula employed in the calculation of these criteria.

**Table 2:** Notation and calculation of the criteria

**Sets**	
*N*:	Set of stroke care networks
*S*_*n*_:	Set of hubs in stroke care network *n*
*P*_*n*_:	Set of spokes in stroke care network *n*
**Parameters**	
*d*_*p*, *s*_:	Driving distance from spoke *p* to hub *s*
${P}_n^{\prime }:$	Number of people supplied by stroke care network *n*
**Formula**	**Criterion description**
$C{1}_n=\frac{\sum_{p\in {P}_n} Mi{n}_{s\in {S}_n}\left\{{d}_{p,s}\right\}}{\left|{P}_n\right|}$	Average travel distance from a spoke to its closest hub within a network *n*, in kilometers
$C{2}_n=\frac{P_n^{\prime }}{\left|{S}_n\right|}$	Average number of inhabitants per hub in network *n*
$C{3}_n=\frac{\left|{S}_n\right|}{\left|{P}_n\right|}$	Ratio of assigned hubs to spokes within network *n*

**Fig. 2:**
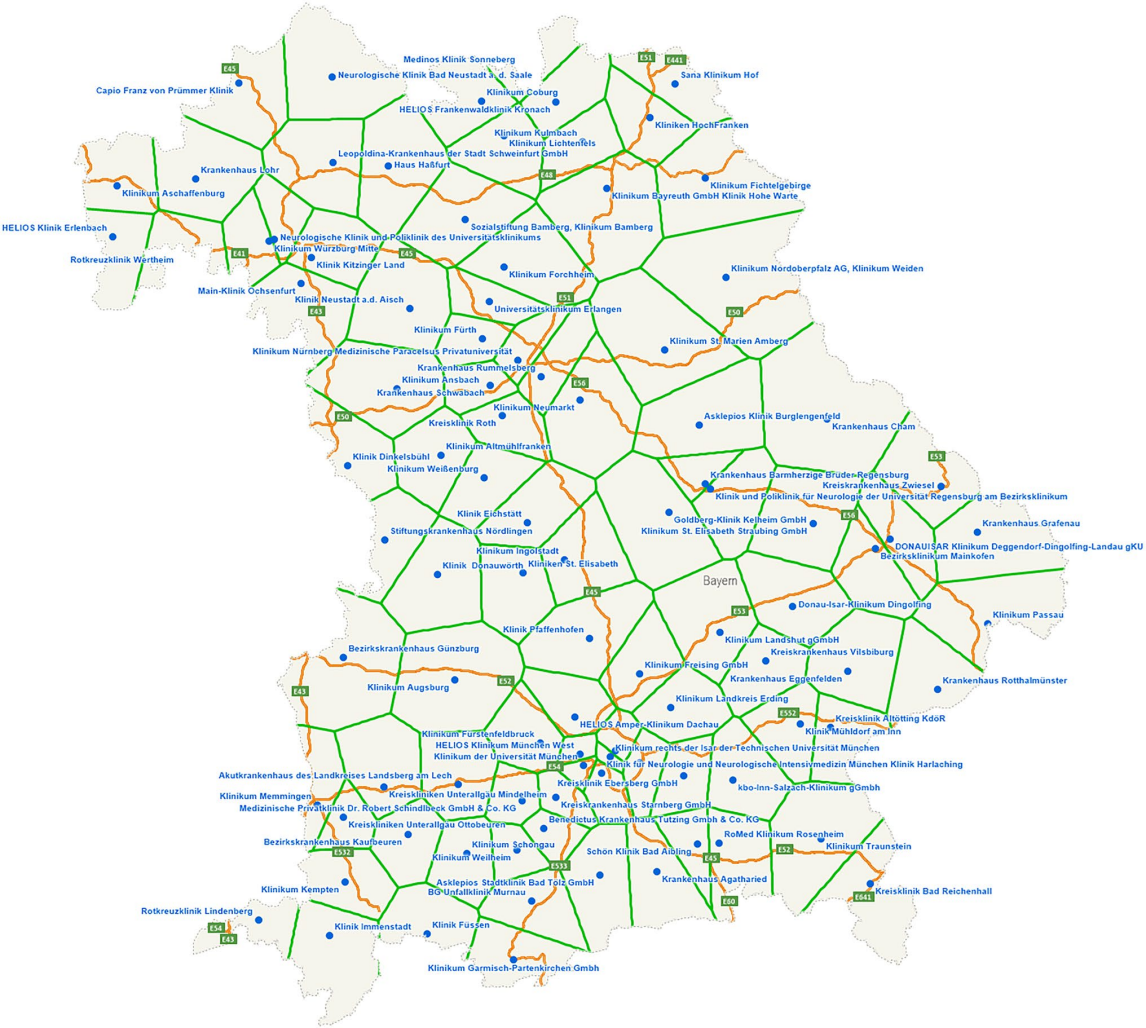
Catchment areas of all 97 hospitals in Bavaria (hospitals are represented by a blue dot, green lines represent the boundaries between catchment areas)

**Fig. 3:**
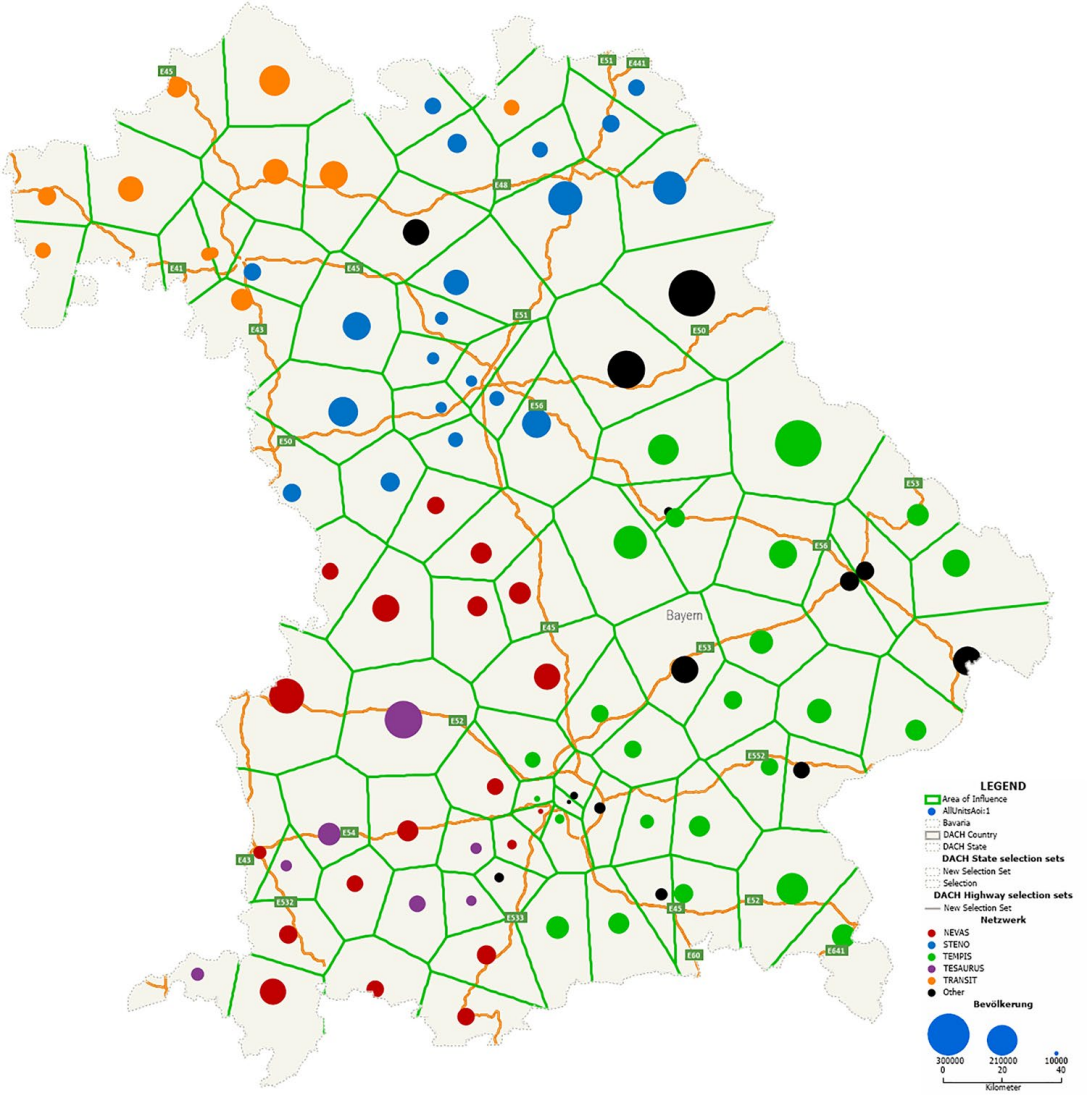
The population of each catchment area

We use Maptitude (Caliper Corporation) to determine the catchment area for each of the 97 hospitals based on their postcode. A postcode belongs to the catchment area of a clinic if the Euclidean distance between its geographical center and this clinic is the smallest. Figure 2 shows these catchment areas. The next step is to determine the number of inhabitants (population) within each catchment area to be served by a hospital. The population data of the 2011 census conducted in Germany are available in Maptitude. Since the census data are available down to the postcode level, the corresponding number of inhabitants was assigned to each postcode area. These figures can then be mapped to the catchment areas. The resulting distribution of the Bavarian population is shown in Fig. 3.

### Assignment of currently independent hubs to stroke care networks

As mentioned before, 14 SUs in Bavaria do not currently belong to any network. We present a mathematical model to assign these SUs to the five existing networks by optimizing (minimizing) the average travel distance to all units within a network. This may increase the ratio of SUs to spoke hospitals, leading to possible improvements. The resulting five networks represent the best possible stroke care network design that could be achieved if the current network design absorbed the independent stroke units. This way, we achieve comparability between the best-case status quo and the optimized green-field type network designs.

**Sets**:

*A*: Set of hubs that do not belong to any stroke care network.

*B* ^*n*^: Set of current hubs and spokes in stroke care network *n* ∈ *N*.

**Parameters**:

*d*_*a*, *b*_: Distance from hub *a* ∈ *A* to spoke *b* ∈ *B*^*n*^

$${m}_{b,n}=\left\{\begin{array}{c}1,\kern0.5em \mathrm{if}\ \mathrm{hospital}\ b\in {B}^n\ \mathrm{belongs}\ \mathrm{to}\ \mathrm{stroke}\ \mathrm{care}\ \mathrm{network}\ n\kern3.25em \\ {}0,\kern0.5em \mathrm{otherwise}\kern13em \end{array}\right.$$

*h*_*n*_: Number of current hospitals in stroke care network *n* ∈ *N*

**Variable**:

$${x}_{a,n}=\left\{\begin{array}{c}1,\kern0.75em \mathrm{if}\ \mathrm{hub}\ a\in A\kern0.5em \mathrm{is}\ \mathrm{added}\ \mathrm{to}\ \mathrm{stroke}\ \mathrm{care}\ \mathrm{network}\ n\in N\kern0.75em \\ {}0,\kern0.75em otherwise\kern12.5em \end{array}\right.$$

**Model:**

1 $${\displaystyle \begin{array}{c}{\operatorname{Min}}\ \sum_{n\in N}\frac{\sum_{a\in A}{\sum}_{b\in {B}^n}{x}_{a,n}\bullet {d}_{a,b}\bullet {m}_{b,n}}{h_n}\\ {}s.t.\end{array}}$$

2 $$\sum_{n\in N}{x}_{a,n}=1,\kern1em \forall a\in A$$

3 $${x}_{a,n}\ is\ binary\ and\in {B}^n,\kern1em \forall a\in A\ and\ n\in N$$

The objective function of the proposed model assigns the currently independent SUs to the existing networks to minimize the average travel distance to all units within the existing network. Equation (2) forces the allocation of each independent SU to exactly one network.

### Clustering

Clustering is usually used in explorative data analysis to divide a given data set into partitions, so-called clusters. In clustering, partitioning into partial data sets is based on the proximity or similarity of characteristics within the data set. The literature contains a large number of clustering problems and algorithms resulting from the different requirements for determining these similarities in the respective models [9]. In cluster analysis, there are various ways of grouping data depending on whether the input variables are qualitative, quantitative, binary, or mixed forms. However, the most intuitive and frequently used decision criterion is the minimum Euclidean distance from the cluster center [12, 35].

The K-Means algorithm has become a standard tool for data analysts in a wide variety of fields [37, 45]. It assigns one of the 𝐾 clusters to a set of *N* points by minimizing the distance, according to the selected distance measure, from each point to the center of the cluster assigned to it [35]. Bayne et al. [7] examined 13 different clustering methods based on various criteria. They concluded that the *K*-means algorithm performed best overall. Therefore, we use the *K*-means clustering method to determine alternatives to the current stroke care network design in Bavaria. The correct value of *K* is often vague and depends on the desired clustering resolution of the user. In fact, the amount of error in the resulting clustering will always keep reducing as we increase *K* without penalty. In this study, we set the maximum number of centers to 5 (i.e., *K* = {2, 3, 4, 5}) to be able to compare the performance of the current networks, which also include five independent networks, to the newly generated networks’ design.

### Simulation

We develop a simulation model to recognize the possible bottlenecks in the stroke care networks in Bavaria as well as to cope with the stochastic arrival rates of patients and the utilization of resources such as monitored stroke beds. First, we explain the important input parameters required for a detailed reflection of stroke care reality in Bavaria. Subsequently, a simulation model is developed using AnyLogic.

#### Arrival time of stroke patients

According to the federal government’s health report in Germany, an average of 420 strokes per 100,000 inhabitants occur annually in Bavaria (Federal Health Report 2018). As Berlis and Weber [8] show, about 80% to 85% of stroke patients have an ischemic cerebral infarction and should be transported to the nearest available hub location. Therefore, we use Eq. (4) to calculate how many strokes occur per 100,000 inhabitants annually in Bavaria.

4 $$\mathrm{a}=\mathrm{x}\ast 0.82$$

where 𝑥 corresponds to the number of strokes per 100,000 inhabitants in Bavaria. 0.825 is the average rate of patients who suffer an ischemic cerebral infarction. Now, we can calculate an arrival rate (*A*_*e*_) for stroke patients in each catchment area (*e* ∈ *E*) using Eq. (5).

5 $${A}_e=\frac{P_e^{\prime }}{100000}\times a$$

Out of these, 5% to 10% are suitable for a mechanical thrombectomy [8]. These patients should be transferred to a stroke center, not a stroke unit. For the current statistics of 420 strokes per 100,000 inhabitants per year from DSG, this results in an approximate occurrence of 26 strokes per 100,000 inhabitants for which an MT in a stroke center is necessary.

#### Length of stay of stroke patients

After admitting stroke patients to a SU or SC, they are treated and stay in the hospital for a certain period in one of the monitored beds. According to the Federal Health Monitoring report in Bavaria, this period ranges from 5.8 to 16.7 days, depending on the type of stroke (including Cerebrovascular diseases [I60-I69] based on the International Statistical Classification of Diseases and Related Health Problems 10th Revision [ICD-10]) from 2000 to 2017. Then, the average length of stay (LOS) is equal to 11.3 days. According to these values, we use a triangular distribution (*trian*~(5.8,11.3,16.7)) to model the patients’ LOS in an SU in the simulated model. The parameters of the triangular distribution can be determined relatively easily and even with little data available [46].

#### Transport routes

Due to the consideration of capacity constraints in the simulation model, a hub hospital may have no free beds available. In this case, the patient(s) would be transported to another hospital in the same network. First, these patients should be transported to the nearest hub in the same network. If there are no free monitored beds, the patients would then be transported to the next nearest hub with free bed capacity. If there is no bed available for the patient in any hub of the network belonging to the catchment area, the patient is forwarded to the closest hub in another network. Then, if there is no available bed in any of the networks, the patient is deemed to be rejected, which is equivalent to non-treatment. This is particularly critical, as an untreated stroke can be fatal or at least result in permanent brain damage.

#### Simulation model

We design a simulation model of the stroke care networks to represent an appropriate compromise between the closest possible proximity to reality and an acceptable runtime. The agent of the simulation is the stroke patient. This represents the victim of a stroke who must be transported from the place of onset to the nearest suitable hub for treatment. The agent simulates the occurrence of a stroke, the selection of an available bed in a hub within the stroke care network at the point of stroke, and the transport to this hub.

**Fig. 4:**
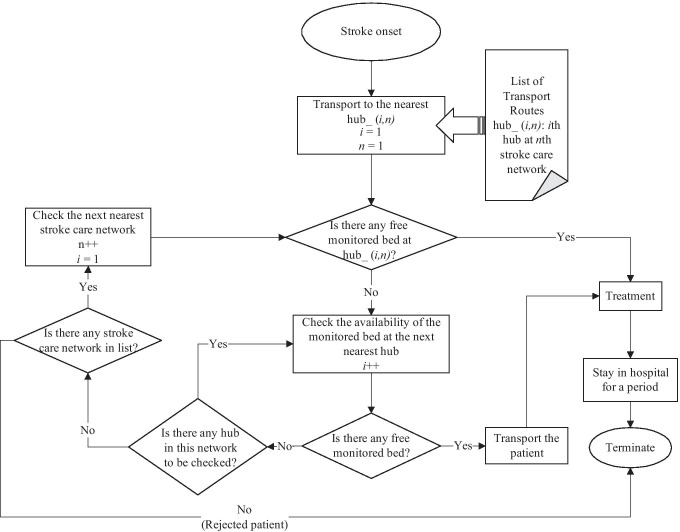
Patient to hub assignment algorithm

The structure of the simulated stroke care network is shown in Fig. 4. Note that our simulation considers two types of patients: Those who can be treated in a SU or a SC, and those who specifically require a mechanical thrombectomy in a SC, as explained in Section 3.3.1. Figure 4 represents the algorithm for the first type of patients, while the logic for the second type of patients is the same, except that only SCs are considered as possible transfer destinations. If a bed is available to the patient in the same network, the patient will move across the map to the nearest hub with an available bed. If there is no free bed in the entire network, the patient is “rejected” from the network and moved to the closest hub with a free bed in another network. Bavaria consists of 97 catchment areas; each of them is represented by an agent in the simulated stroke care network model. Patients who are taken from a point of a stroke to a hub are held there for the duration of their stay and then discharged. After patients are discharged, they are not considered further and are therefore removed from the simulation process. In order to visualize the travel routes of the patients and the network members, the simulation also includes a map of Bavaria on which the patients move according to the rules described above. The model serves to determine the distances a patient travels from the point of a stroke to the assigned hub. We use AnyLogic’s board tools to incorporate the road infrastructure of Bavaria to determine the best (shortest) way for transporting patients. The warmup period of the simulation is two months, after which each simulation run takes one year. For each evaluated network configuration, we conduct 1000 independent runs. Every run starts with a new random seed that is the same for all network configurations.

#### Simulation validation

We validated our simulation results using both historical data validation and face validity. Having access to two years of patient data from one of the stroke centers involved in our study, we can compare the frequency of patient arrivals and bed utilization levels from our simulation study to the real-world data. We aggregate patient data from the stroke center to monthly averages over two years, resulting in 24 observations over 2 years. The results of the t-Test with the null hypothesis *H*_0_ : *μ*_*real*_ = *μ*_*sim*_ against the alternative hypothesis *H*_1_ : *μ*_*real*_ ≠ *μ*_*sim*_ are reported in Table 3 and suggest that our simulation results are very close to the real data, as the null hypothesis that the means are equal cannot be rejected at a significance level of 5%. From the result of the t-Test, we retain the null hypotheses (i.e., equality of means) for both performance measures.

**Table 3:** Results of t-Test for simulation validation

	Monthly patient arrivals		Bed utilization	
	*Real System*	*Simulated System*	*Real System*	*Simulated System*
Mean	169.292	163.667	82.250	83.792
Variance	172.476	169.710	19.848	20.520
t Stat	1.453	–	−1.080	–
p value	0.160	–	0.291	–

Moreover, publicly available reports (in German) from the stroke care networks provide some aggregate information on treated patients that allowed us to further check our results. Finally, we discussed our results with an expert involved in managing one of the stroke care networks.

## Results and discussions

This section presents the results of each stage of the proposed framework and provides answers to our research questions.

### Performance of current stroke care networks design

We calculate the three performance criteria for the current structure of stroke care networks (scenario 0). To this end, we use the data provided by DSG and present the results in Table 4 as scenario 0. The results indicate that STENO is the best performing stroke care network with respect to all three criteria. A small number of people to be treated compared to the other stroke care networks and the high ratio of hubs to spokes are particularly striking. TEMPiS is in second place. It serves almost twice as many people per hub as STENO and has a much lower hub-to-spoke ratio compared to STENO. NEVAS and TRANSIT are third and fourth. TESAURUS is last concerning all criteria. The average distance of the spokes to the closest hub in the TESAURUS network is almost three times as high as in the STENO network and almost twice as high as the overall closest-distance-to-hub average. While TESAURUS has to serve about five times as many inhabitants per hub as STENO and more than twice as many as the average, its hub-to-spoke ratio is significantly lower than in the other networks.

**Table 4:** Performance criteria calculated for scenarios

Scenario	Method	Networks	Number of members	Performance criteria		
				C1 (Average travel distance in km)	C2 (Population to hubs ratio in thousands)	C3 (Hub to spoke ratio)
0	Current design	TEMPiS	24	46.59	270.79	1.18
		STENO	21	29.29	153.85	4.25
		NEVAS	19	35.19	295.14	0.73
		TRANSIT	12	48.70	312.05	0.71
		TESAURUS	7	92.93	779.10	0.17
			Weighted Average *	**43.82**	**-**	**-**
0+	After assignment of the independent SUs	TEMPiS+	33	33.23	202.34	2.00
		STENO+	24	29.18	169.58	5.00
		NEVAS	19	35.19	295.14	0.73
		TRANSIT	12	48.70	312.05	0.71
		TESAURUS+	9	65.85	279.92	0.50
			Weighted Average	**37.55**		
1	Single network	Single-Network	Weighted Average	**30.48**	**217.31**	**1.49**
2	Clustering with k=2	1	38	32.69	206.65	1.92
		2	59	32.54	225.40	1.27
			Weighted Average	**32.60**	**-**	**-**
3	Clustering with k=3	1	39	35.46	214.62	1.60
		2	29	33.13	253.34	1.64
		3	29	26.43	180.83	1.23
			Weighted Average	**32.06**	**-**	**-**
4	Clustering with k=4	1	27	34.35	248.80	1.70
		2	23	27.11	183.99	0.92
		3	21	28.19	194.91	2.50
		4	26	36.80	228.48	1.36
			Weighted Average	**31.96**	**-**	**-**
5	Clustering with k=5	1	21	28.72	211.03	2.00
		2	12	30.15	214.02	3.00
		3	26	34.35	243.51	1.60
		4	26	26.43	173.52	1.00
		5	12	37.28	261.96	1.00
			Weighted Average	**30.85**	**-**	**-**

*The averages for C1 are weighted by the number of SUs in each network

### Assignment of the independent stroke units

**Fig. 5:**
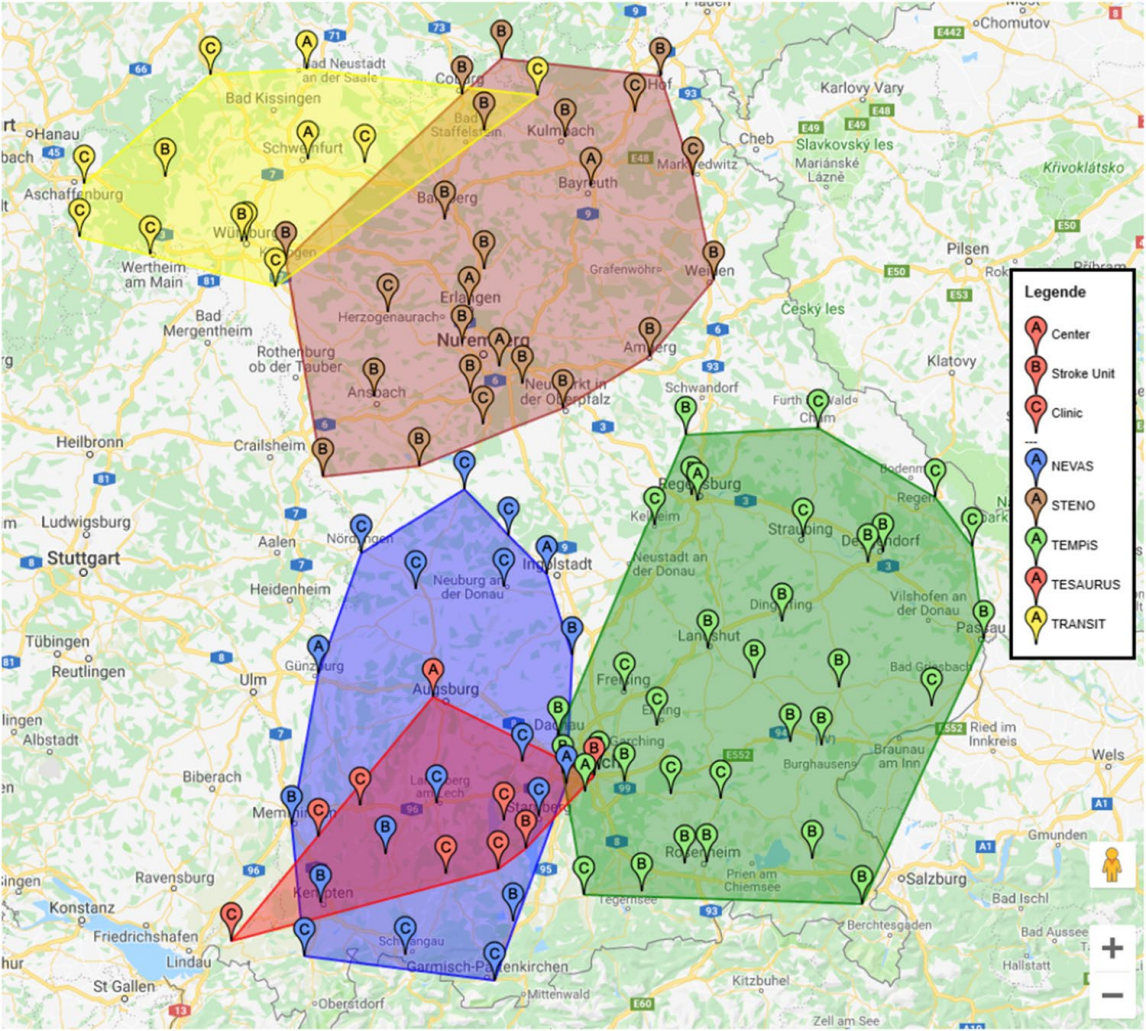
Stroke care networks after the allocation (scenario 0+)

After running the proposed mathematical model, 14 independent SUs are assigned to scenario 0 of Bavaria. Three of these SUs are assigned to the STENO, 9 SUs to the TEMPiS, and 2 to the TESAURUS networks, respectively. TRANSIT and NEVAS do not receive any new SUs. A visual representation of the stroke care networks after the allocation of new SUs (scenario 0+) is shown in Fig. 5.

The results in Table 4 indicate that after the assignment of the independent SUs to the current stroke care network design, the key performance criteria of the networks have changed. NEVAS and TRANSIT remain unchanged since no new SU is added to them. In the STENO network, the newly added SUs lead to a slight decrease in the average distance from a hospital to the nearest SU as well as an increase in C2. The ratio of hubs to hospitals without corresponding facilities is also increased in the new design. A similar situation can be seen in the TEMPiS network but to a greater extent. The most significant changes occur in TESAURUS, where the average distance from a spoke to a hub is reduced by 29%. The value of C2 decreases from 779,105 to 279,916, which corresponds to a reduction of 64.07%. We can also see similar differences in C3. The improvements of scenario 0+ compared with scenario 0 are reflected in the weighted average value as well. For C1, the weighted average distance to be covered is reduced by 6.27 km across all stroke care networks. Scenario 0+ examined here proves to be superior to scenario 0 concerning all criteria. The reason for this can be found in the fact that strategically more favorable locations are added to the networks. Furthermore, these new network members remove the spatial and structural limitations of the previous network boundaries.

### Scenarios designed based on clustering

**Fig. 6:**
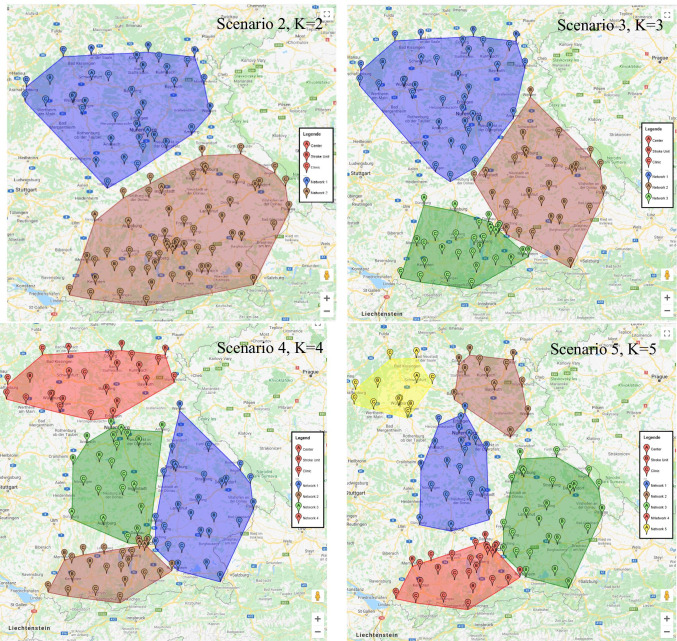
New scenarios (stroke care network designs) generated by K-Means

We utilize K-means to design 4 more scenarios (*K* = {2, 3, 4, 5}) and evaluate the performance of these new scenarios as shown in Fig. 6 with the help of the defined criteria. Since K-Means only finds a local optimum, K-Means is performed 100 times in the first step. In a further step, those cluster solutions in which there are empty networks, i.e. those with no members, are excluded from these cluster solutions. Thus for *K* = 2 and *K* = 3, there are 10 and 20 unique, non-empty solutions, respectively. At *K* = 4 and *K* = 5, there are 43 and 48 solutions left, in turn. We report the results of the best respective solution concerning criterion C1 for each number of generated networks.

Now, in order to make a statement about improvements or deteriorations of the scenarios compared to scenario 0 and each other, we calculate the value of performance criteria for the new scenarios as well. The results of all scenarios are summarized in Table 4.

In network design, there are no restrictions on forming a single large network in which each stroke unit can be connected to each hospital. To this end, we combine all hubs and spokes from all networks into a single network and call it scenario 1. With this scenario, the average distance to the nearest hubs is 13 (7) kilometers shorter than scenario 0 (0+). Moreover, we find that the currently existing network design is inferior to both the approach of including the independent stroke units and the single-network solution. To assess the performance of these two alternatives, we can compare them with the best solutions of scenarios 2 through 5.

The results of clustering indicate that the relative range of the number of members between networks decreases as the number of networks increases. With respect to C1, we find that the network designs that were determined through our clustering approach outperform the current design and scenario 0+. Not only are the weighted average C1 values considerably lower, but there is also a much better balance between the individual networks within each new design. Hence, the maximum average travel distance of the worst-performing network is much lower in each of the new scenarios compared to scenarios 0 and 0+, which results in shorter transfers and better treatment outlooks for patients that are admitted to a hospital within these networks. Likewise, the population-to-hubs ratio is much more balanced in scenarios 1 through 5, reducing the potential overload of a single network, especially compared to the population of 779,100 that the TESAURUS network currently serves with its single hub. Note that this balance is achieved even though our clustering approach focuses on finding the best network designs concerning only the average travel distance. The hub-to-spoke ratios of the individual networks within each design also become much less variable. Therefore, the new designs lower the potential for capacity bottlenecks at the hub locations and decrease the chance that a patient cannot be accommodated within the network. This again indicates that we find much more balanced network designs than the one currently existing in Bavaria. There is no clear optimal number of networks, but we can conclude that all clustering-based network designs are better than the network structures in scenarios 0 and 0+ concerning the defined criteria.

### Results of simulation modeling

The performance of the two best stroke care network designs, i.e., scenarios 1 and 5 are analyzed via the simulation. First, the current network structure (scenario 0) with its 5 networks and 14 independent SUs is simulated. The aim is to check whether the current system can meet the patient demand requirements. The second scenario is the single-network design. We examine whether there must be frequent transports to more distant hubs without restrictions by network borders to treat every patient. Scenario 5 with five networks (*K* = 5) is also simulated to conduct a comprehensive comparison with the current situation (scenario 0). We investigate whether a solution that offers both parties - patients and hospitals - a middle course between short travel distances and economic profitability - can be a sensible alternative to the existing network design. The parameter analysis presented is performed for each scenario with 1000 replications. They are evaluated based on four criteria containing SU bed utilization, the number of patients rejected to another network, the proportion of patients diverted within the same network, and the average traveled distance per rejected or diverted patient in kilometers. We present the results obtained from the simulated model in Table 5.

**Table 5:** Results of scenario analysis obtained from the simulation

Scenario	Network	SU/SC Bed Utilization		Number of patients rejected to another network per year		Proportion of patients diverted (within network)		Avg. traveled distance per rejected or diverted patient (in km)	
		Mean	StdDev	Mean	StdDev	Mean	StdDev	Mean	StdDev
0	NEVAS	72%	6.1%	0.07	0.20	14.8%	4.0%	19.6	5.7
	STENO	66%	4.0%	0	0	4.2%	0.6%	4.6	1.3
	TEMPiS	78%	7.1%	0	0	7.5%	1.2%	24.3	6.6
	TESAURUS	84%	9.5%	0.12	0.32	-	-	58.7	0
	TRANSIT	71%	5.7%	0.09	0.28	6.6%	1.0%	23.4	4.2
	Unassigned SUs	61%	11.2%	0.23	0.43	-	-	3.4	2.3
	Weighted average*	**75%**	**-**	**0.08**	**-**	**8.7%**	**-**	**18.3**	**-**
1	Single-network	**72%**	**5.0%**	**0**	**-**	**6.8%**	**1.3%**	**10.7**	**3.8**
5	1	69%	5.4%	0	0	7.1%	1.1%	9.1	3.0
	2	68%	4.2%	0	0	5.2%	0.9%	6.5	1.5
	3	72%	4.1%	0	0	7.9%	1.1%	11.6	3.2
	4	73%	3.9%	0	0	7.2%	1.3%	12.4	3.3
	5	78%	8.3%	0	0	8.7%	1.6%	19.2	6.1
	Weighted average*	**73%**	**-**	**0**	**-**	**7.6%**	**-**	**11.2**	**-**

*The averages are weighted by the number of SUs in each network except for the avg. traveled distance, which is the sum of all traveled distances by diverted or rejected patients divided by the number of diverted or rejected patients

#### Utilization of stroke units

The utilization of an SU can have a decisive influence on treatment quality. High utilization may lead to errors more frequently due to the strain on the personnel. The overall (weighted) averages for each network design are quite similar, as the randomly generated patient admissions and lengths of stay are the same for each network configuration. Interestingly, the variability of the SU bed utilization in the different networks differs considerably between scenarios 0 and 5. In scenario 0, for example, the mean utilization of the STENO network is 66%, whereas the TESAURUS network has a mean utilization of 85%. In scenario 5, the mean utilizations range between 68% and 78%. This indicates that scenario 5 offers a more balanced supply of SUs and SCs, which is also supported by the overall lower standard deviations across the simulation runs.

#### Number of patients rejected to another network

The number of patients rejected to another network per year describes those patients who are not assigned to a bed in an SU/SC within the same network. These patients do not receive the quickest possible treatment in an SU, which can have serious consequences for their recovery. Such cases may occur in the event of the sudden arrival of several patients in a short period of time. In the current network design (scenario 0), there are three networks plus the unassigned SUs where patients may be rejected – even if only in a few cases. The weighted average number of rejected patients per year is 0.08 in this network configuration. Even if this number is negligibly small, the number of stroke occurrences is likely to increase in the future. This can lead to bottlenecks in the current network design. At the same time, the numbers for the independent SUs are even higher. Here, the capacity becomes a bottleneck more frequently, leading to patient transfers to another hospital without the benefit of established within-network communication and information technology. In scenario 5, there are no rejected patients. Thus, each arriving stroke patient can be assigned to a monitored bed within the respective network in all simulation runs. In conclusion, scenario 5 outperforms scenario 0 concerning the average number of patients that need to be moved outside of the network.

#### Proportion of patients that are diverted

How often a patient should be diverted to the next-closest hub within a network is also important in stroke care network design. This results in longer transportation times for the patients and thus a delay in their treatment. For the medical staff, this results in an additional organizational effort to find a SU/SC for the patient. Thus, the lower the proportion of patients that need to be diverted, the better a network performs. In scenario 0, the independent SUs and TESAURUS cannot divert patients (as TESAURUS only has one hub). The most undesirable result can be found in the NEVAS network in the current network design, where almost every sixth patient (14.8% on average) has to be diverted. Apart from that, the different network designs perform quite similarly, with the exception that scenario 5 again shows less variability across the different networks.

#### Traveled distance

As with the number of diversions that patients face on the way to an SU/SC, the distance they travel to such a facility must be kept as low as possible to treat them as soon as possible. In addition to the capacity bottlenecks already described, the structure of the individual networks can also play a decisive role here since they represent the geographical limitation of the routes in the current network design. The results show that the average distance a diverted or rejected patient has to travel for treatment is considerably higher in scenario 0 (18.3 km) than in the other two scenarios (10.7 km and 11.2 km, respectively). While in STENO, a stroke patient has to be transported less than 5 km on average, the value for the other networks is significantly higher. The TESAURUS network, in particular, will need to transfer patients almost 60 km on average once its single hub cannot take on a new patient due to insufficient capacity. Based on these values, it can be concluded that the single-network design is superior to the current network design (scenario 0), while scenario 5 does not trail far behind. Similar to the other evaluations, this analysis shows that the current network design has a lot of improvement potential if more attention is paid to travel distances.

#### Discussion

While the current network design is fairly competitive in terms of capacity utilization except for its lack of balance between the individual networks, it falls behind the proposed alternatives in the other indicators. Therefore, one of the two alternatives to the current stroke care network design would be preferable. The main difference is that the single-network design achieves better outcomes, while scenario 5 provides more balanced results among all other network designs. The control and management of the single network depend upon all of the hospitals, and it is difficult for one given SU/SC to differentiate itself.

However, it should be noted that the current network design can by no means be considered a failure. Even if travel distances could be shortened, there are hardly any occurrences in which patients have to be rejected from the networks. However, the independent SUs have a higher number of rejected patients per year than those who are part of a network, despite their low average capacity utilization. This indicates seasonal fluctuations leading to increased patient numbers within short periods, which leads to patient rejections. It may be sensible for SUs that do not belong to a network to increase the stroke care capacities to avoid the few occurrences of patient rejections after weighing up the costs and benefits. This would be desirable for the benefit of the patient as well.

Demographic development plays an essential role in stroke care. It is therefore crucial for the network structures in Germany to keep a long-term eye on demographic changes to identify potential bottlenecks in the current structure at an early stage and to implement structural improvements that are also economically viable. From the patients’ point of view, it is advisable to restructure the networks. Stroke care networks are still to be regarded as sensible, as many of the previously discussed advantages support. The aim here is to find a solution that is acceptable for both sides, which benefits the patients and at the same time allows the hospitals to remain profitable.

## Conclusions

The use of telestroke and stroke care networks has significantly expanded over the past decade. The fact that stroke therapy is a time-critical disease process, coupled with the relative scarcity of resources, makes the establishment of stroke care networks an attractive approach. In this line, we propose an approach to identify different network structures with a focus on patient transfer travel distances. We consider alternative designs for the current stroke care networks and SUs without affiliation in Bavaria, Germany. These designs are evaluated in a simulation study, which allows us to consider stochasticity in demand as well as the limited bed capacity, and their performance is compared to the current system. The two most interesting alternatives outperform the current stroke care network in Bavaria in almost every aspect.

The results emphasize the areas of development and potential improvement of stroke care network designs and illustrate how their performance can be improved. The results also demonstrate that independent SUs have a higher number of rejected patients than those that are a member of a stroke care network. As already stated in the motivation, demographic development plays an essential role in stroke care. It is therefore essential for the network structures in Germany to keep a long-term eye on population change. Only in this way can scenarios that would lead to supply bottlenecks with the current structure be identified at an early stage, and structural improvements that are also economically viable can be implemented.

From the patients’ perspective, there is room for improvement for the current network design of the stroke care networks in Bavaria. However, it should be noted that this study entails limitations, which are described in the following. Subsequently, an outlook is given on further research possibilities in this area.

One limitation lies in the scarcely available input data used for simulation. The first step is to refine the patient data generation. In our simulation model, these are only roughly determined and do not differ except for the population density of the individual catchment areas. It is advisable to include further demographic data. Since the probability of a stroke also depends on the age of the person affected, this distribution must be included in the determination of the arrival rate. In addition to data on demographic change, additional data collection is needed for seasonal variations in stroke. The inclusion of these factors makes it possible to determine arrival rates much more accurately. Moreover, the aspect of economic efficiency should not be neglected. In the present study, the focus is on the care of patients. SUs, in particular, are confronted with financial losses due to the expansion of networks by establishing further SUs because the stroke patients are then distributed among more stroke centers.

Nevertheless, the concept of stroke care networks should be critically questioned. What was initially conceived as supra-regional, rapid, and competent medical support has shifted into a mostly economic construct. The telemedicine networks have their reasons for the exchange of knowledge, expert advice across hospitals, and consultation of experts. Today, it is easy to consult experts whose location is almost irrelevant when diagnosing a patient. The consultant role should be decoupled from the physical delivery of a patient to the SU.

## Footnotes

1 In German: Gesundheitsberichterstattung des Bundes

2 https://www.dsg-info.de/

## References

[1] Ahmadi-Javid A, Seyedi P, Syam SS (2017). A survey of healthcare facility location. Comput Oper Res.

[2] Aringhieri R, Bruni ME, Khodaparasti S, van Essen JT (2017). Emergency medical services and beyond: addressing new challenges through a wide literature review. Comput Oper Res.

[3] Audebert H (2006). Telestroke: effective networking. The Lancet Neurology.

[4] Audebert HJ, Lichy C, Szabo K, Schäbitz WR (2008). Telemedizin und Stroke Unit. Notfall + Rettungsmedizin.

[5] Audebert HJ, Schultes K, Tietz V, Heuschmann PU, Bogdahn U, Haberl RL, Schenkel J (2009). Long-term effects of specialized stroke care with telemedicine support in community hospitals on behalf of the Telemedical project for integrative stroke care (TEMPiS). Stroke.

[6] Bavarian State Ministry of Health and Care (2020) https://www.stmgp.bayern.de/meine-themen/fuer-krankenhausbetreiber/telemedizin/#Projekte-in-Bayern. Accessed 5 June 2019

[7] Bayne CK, Beauchamp JJ, Begovich CL, Kane VE (1980). Monte Carlo comparisons of selected clustering procedures. Pattern Recogn.

[8] Berlis A, Weber W (2017). Flächendeckende Akutversorgung von Schlaganfallpatienten durch die (Neuro) -Radiologie ist gewährleistet. Fortschr Röntgenstr.

[9] Boujelben MA (2017). A unicriterion analysis based on the PROMETHEE principles for multicriteria ordered clustering. Omega.

[10] Churilov L, Donnan GA (2012). Operations research for stroke care systems: an opportunity for the science of better to do much better. Operations Research for Health Care.

[11] Cossey TC, Jagolino A, Ankrom C, Bambhroliya AB, Cai C, Vahidy FS, Savitz SI, Wu T-C (2019). No weekend or after-hours effect in acute ischemic stroke patients treated by telemedicine. J Stroke Cerebrovasc Dis.

[12] de Smet Y, Nemery P, Selvaraj R (2012). An exact algorithm for the multicriteria ordered clustering problem. Omega.

[13] Etherton MR, Schwamm LH (2018). Telestroke for the newly minted vascular neurologist. Stroke.

[14] Fanale CV, Demaerschalk BM (2012). Telestroke network business model strategies. J Stroke Cerebrovasc Dis.

[15] Feil K, Rémi J, Küpper C, Herzberg M, Dorn F, Kunz WG, Reidler P, Levin J, Hüttemann K, Tiedt S, Heidger W, Müller K, Thunstedt DC, Dabitz R, Müller R, Pfefferkorn T, Hamann GF, Liebig T, Dieterich M, Kellert L (2020) Inter-hospital transfer for mechanical thrombectomy within the supraregional stroke network NEVAS. J Neurol:1–9 10.1007/s00415-020-10165-2 PMC7880976 32889616

[16] French B, Day E, Watkins C, McLoughlin A, Fitzgerald J, Leathley M, Davies P, Emsley H, Ford G, Jenkinson D, May C, O’Donnell M, Price C, Sutton C, Lightbody C (2013). The challenges of implementing a telestroke network: a systematic review and case study. BMC Med Inform Decis Mak.

[17] Gesundheit in Deutschland (2015) p. 45. Kapitel 02: Wie steht es um unsere Gesundheit? Einzelkapitel aus "Gesundheit in Deutschland 2015"; 2015. https://www.rki.de/DE/Content/Gesundheitsmonitoring/Gesundheitsberichterstattung/GBEDownloadsGiD/2015/02_gesundheit_in_deutschland.pdf?__blob=publicationFile. Accessed 3 June 2019

[18] Hess DC, Audebert HJ (2013) The history and future of telestroke. Nat Rev Neurol 2013;9; 340 EP - 10.1038/nrneurol.2013.86 23649102

[19] Hubert GJ, Meretoja A, Audebert HJ, Tatlisumak T, Zeman F, Boy S, Haberl RL, Kaste M, Müller-Barna P (2016). Stroke thrombolysis in a centralized and a decentralized system (Helsinki and Telemedical project for integrative stroke care network). Stroke.

[20] Hubert GJ, Santo G, Vanhooren G, Zvan B, Tur Campos S, Alasheev A, Abilleira S, Corea F (2018). Recommendations on telestroke in Europe. European Stroke Journal.

[21] Imai T, Sakurai K, Hagiwara Y, Mizukami H, Hasegawa Y (2014). Specific needs for Telestroke networks for thrombolytic therapy in Japan. J Stroke Cerebrovasc Dis.

[22] Jauch EC, Saver JL, Adams HP, Bruno A, Connors JJB, Demaerschalk BM, Khatri P, McMullan PW, Qureshi AI, Rosenfield K, Scott PA, Summers DR, Wang DZ, Wintermark M, Yonas H (2013). Guidelines for the early management of patients with acute ischemic stroke: a guideline for healthcare professionals from the American Heart Association/American Stroke Association. Stroke.

[23] Keshtkaran M, Churilov L, Hearne J, Abbasi B, Meretoja A (2016). Validation of a decision support model for investigation and improvement in stroke thrombolysis. Eur J Oper Res.

[24] Khan K, Shuaib A, Whittaker T, Saqqur M, Jeerakathil T, Butcher K, Crumley P (2010). Telestroke in northern Alberta: a two year experience with remote hospitals. Canadian Journal of Neurological Sciences / Journal Canadien des Sciences Neurologiques.

[25] Kraft P, Hillmann S, Rücker V, Heuschmann PU (2017). Telemedical strategies for the improvement of secondary prevention in patients with cerebrovascular events—a systematic review and meta-analysis. Int J Stroke.

[26] Lampert T, Müters S, Kuntz B, Dahm S, Nowossadeck E (2019). 30 years after the fall of the Berlin Wall: regional health differences in Germany. Journal of Health Monitoring.

[27] Langhorne P, Dennis MS (2004). Stroke units: the next 10 years. Lancet.

[28] Langhorne P, Bernhardt J, Kwakkel G (2011). Stroke rehabilitation. Lancet.

[29] Levine Steven R, Gorman Mark. “Telestroke” the application of telemedicine for stroke. Stroke 1999;30; 464–469 10.1161/01.str.30.2.464 9933289

[30] Meyer BC, Demaerschalk BM (2012). Telestroke Network Fundamentals. J Stroke Cerebrovasc Dis.

[31] Müller-Barna P, Hubert GJ, Boy S, Bogdahn U, Wiedmann S, Heuschmann PU, Audebert HJ (2014). TeleStroke units serving as a model of care in rural areas: 10-year experience of the TeleMedical project for integrative stroke care. Stroke.

[32] Nelson RE, Saltzman GM, Skalabrin EJ, Demaerschalk BM, Majersik JJ (2011). The cost-effectiveness of telestroke in the treatment of acute ischemic stroke. Neurology.

[33] Nimptsch U, Mansky T (2012). Trends in acute inpatient stroke care in Germany--an observational study using administrative hospital data from 2005-2010. Deutsches Arzteblatt international.

[34] Pallesen LP, Winzer S, Barlinn K, Prakapenia A, Siepmann T, Gruener C, Gerber J, Haedrich K, Linn J, Barlinn J, Puetz V (2020). Safety of inter-hospital transfer of patients with acute ischemic stroke for evaluation of endovascular thrombectomy. Sci Rep.

[35] Peng J, Wei Y (2007). Approximating K-means-type clustering via semidefinite programming. SIAM J Optim.

[36] Pidd M (2010). Why modelling and model use matter. J Oper Res Soc.

[37] Sarrazin R, de Smet Y, Rosenfeld J (2018). An extension of PROMETHEE to interval clustering. Omega.

[38] Saver JL (2006). Time is brain--quantified. Stroke.

[39] Deutsche Schlaganfall-Gesellschaft. Liste der zertifizierten Stroke Units in Deutschland (2018) https://www.dsg-info.de/stroke-units/stroke-units-uebersicht.html. Accessed 5 June 2019

[40] Schwamm LH, Rosenthal ES, Hirshberg A, Schaefer PW, Little EA, Kvedar JC, Petkovska I, Koroshetz WJ, Levine SR (2004). Virtual TeleStroke support for the emergency department evaluation of acute stroke. Acad Emerg Med.

[41] Susman E (1997). Telemedicine to give rural stroke victims fair chance of recovery with new treatment. Telemedicine Virtual Reality.

[42] Switzer JA, Demaerschalk BM (2012). Overcoming challenges to sustain a Telestroke network. J Stroke Cerebrovasc Dis.

[43] Switzer JA, Hall CE, Close B, Nichols FT, Gross H, Bruno A, Hess DC (2010). A Telestroke network enhances recruitment into acute stroke clinical trials. Stroke.

[44] The Federal Health Monitoring System (2015) Cost of illness in millions of Euro for Germany. Classification: years, sex, ICD10, type of facility. Table (ad hoc): Cost of illness by facility, sex, ICD10 (from 2015); 2015. http://www.gbe-bund.de/oowa921-install/servlet/oowa/aw92/dboowasys921.xwdevkit/xwd_init?gbe.isgbetol/xs_start_neu/&p_aid=i&p_aid=20746038&nummer=64&p_sprache=E&p_indsp=-&p_aid=96069248#SOURCES. Accessed 5 June 2019

[45] Wagstaff K, Cardie C, Rogers S, Schrödl S (Eds) (2001) Constrained k-means clustering with background knowledge

[46] Wing CK (1995). The validity of the triangular distribution assumption in Monte Carlo simulation of construction costs: empirical evidence from Hong Kong. Constr Manag Econ.

[47] World Health Organization (2017) Fact Sheet - Cardiovascular Diseases. https://www.who.int/en/news-room/fact-sheets/detail/cardiovascular-diseases-(cvds). Accessed 12 April 2020

[48] Zerna C, von Kummer R, Gerber J, Engellandt K, Abramyuk A, Wojciechowski C, Barlinn K, Kepplinger J, Pallesen L-P, Siepmann T, Dzialowski I, Reichmann H, Puetz V, Bodechtel U (2015). Telemedical brain computed tomography misinterpretation by stroke neurologists is not associated with thrombolysis-related intracranial hemorrhage. J Stroke Cerebrovasc Dis.

[49] Zerna C, Jeerakathil T, Hill MD (2018). Telehealth for remote stroke management. Can J Cardiol.

